# A Comprehensive Review of Personal Care Aide Training Standards Across the United States

**DOI:** 10.1093/geroni/igaf122.763

**Published:** 2025-12-31

**Authors:** Jessica King, Kezia Scales

**Affiliations:** PHI, New York, New York, United States; PHI, New York, New York, United States

## Abstract

Personal care aides (PCAs), unlike other direct care workers, are not subject to any federal minimum training requirements. Therefore, PCA training standards vary across states. This study aimed to generate a comprehensive analysis of state-by-state PCA training standards, updating and extending a 2014 analysis. To accomplish this, we conducted a content analysis of state Medicaid manuals, waiver documents, and administrative code pertaining to home care programs and services. We assessed the requirements according to three domains: consistency (whether the training requirements were consistent across Medicaid programs); rigor (according to indicators such as named competencies and minimum training hours, among others); and portability (whether training is tied to a recognized certification and/or centrally recorded on a registry), as well as whether states had training requirements for independent providers and private-pay PCAs. We identified six “leader states” who scored highly across all domains: Washington D.C., New Jersey, New York, Oregon, Rhode Island, and Washington State. We found that 65 percent of states (33 states) had consistent training requirements across all Medicaid programs, while 14 percent (7 states) did not have any requirements. This patchwork of state training standards has implications for workforce preparedness and mobility, recruitment and retention, and care quality. The study findings can help state leaders assess the adequacy of their training standards, learn from other states, and implement changes, working toward a goal of building a stable, competent workforce to care for older adults and people with disabilities.

